# Bifocal retinal degeneration observed on ultra-widefield autofluorescence in some cases of *CRX*-associated retinopathy

**DOI:** 10.1038/s41433-024-03522-2

**Published:** 2024-12-04

**Authors:** Siying Lin, Gavin Arno, Anthony G. Robson, Elena R. Schiff, Moin D. Mohamed, Michel Michaelides, Andrew R. Webster, Omar A. Mahroo

**Affiliations:** 1 National Institute of Health Research Biomedical Research Centre at Moorfields Eye Hospital and The UCL Institute of Ophthalmology, London, UK; 2 UCL Institute of Ophthalmology, University College London, London, UK; 3 Greenwood Genetic Center, Greenwood, SC USA; 4 Department of Ophthalmology, St Thomas’ Hospital, London, UK

**Keywords:** Disease genetics, Eye manifestations, Retinal diseases

## Abstract

**Background:**

Variants in *CRX* are associated with dominantly inherited retinopathy with considerable phenotypic variability. Many patients have central retinal degeneration; in some patients, we have observed an additional focus of degeneration in the nasal retina. This study explores this phenotypic association amongst patients with *CRX*-associated disease.

**Methods:**

A retrospective review was conducted for all patients with dominant *CRX*-associated retinopathy at two UK centres. Analysis focused on patients with available ultra-widefield autofluorescence imaging and aimed to identify those with a specific bifocal degeneration pattern involving the nasal retina in both eyes.

**Results:**

Sixty patients were identified, with ultra-widefield fundus imaging available for 50 patients. Of these, six male patients aged 26–74 years displayed a distinct pattern characterised by central retinal degeneration and an additional discrete area of altered autofluorescence in the nasal periphery. Pattern and full-field ERGs indicated macular dysfunction in all 6 cases, with generalised cone (*n* = 2) or cone and rod (*n* = 4) system involvement, with a locus that appeared to be post-phototransduction. The *CRX* variants found in these patients included missense variants (*n* = 2), frameshifting variants (*n* = 3), and a *CRX* whole gene deletion (*n* = 1), with no clear genotype-phenotype correlation identified.

**Conclusions:**

We report a distinct pattern of bifocal retinal degeneration in some cases of *CRX*-associated retinopathy (12% in our cohort), not typically seen in other forms of inherited retinal disease. Recognising such phenotypes can guide genetic investigations or their interpretation, facilitating molecular diagnoses for effective family counselling, given the autosomal dominant inheritance and phenotypic variability of *CRX*-associated retinopathy.

## Introduction

The cone rod homeobox-containing gene (*CRX*; MIM *602225), located on chromosome 19q13.33, encodes a transcription factor essential for both photoreceptor development and survival [1–3]. Pathogenic *CRX* variants are associated with a range of retinal dystrophy phenotypes, including macular dystrophy [4], cone-rod dystrophy (MIM #120970) and Leber congenital amaurosis (MIM #613829) [5]. Over 150 disease-causing *CRX* variants have been reported, including missense, nonsense, frameshift and splice site variants, as well as multi-exon and whole gene deletions (The Human Gene Mutation Database® Professional 2023.4, accessed on 28 March 2024), most commonly causing dominant disease. In rare cases, biallelic variants in *CRX* have been reported in association with autosomal recessive Leber congenital amaurosis [6].

Dominant *CRX*-associated retinopathy exhibits considerable phenotypic variability, even within families sharing the same mutant *CRX* allele. Degeneration affecting the central or paracentral retina tends to be a consistent feature, with variable degrees of wider retinal involvement. In some patients, we have observed an additional, non-contiguous focus of degeneration in the nasal retina. This study aims to describe this previously unreported association in a series of patients with dominant *CRX-*associated retinopathy, and to explore the prevalence of such a pattern.

## Methods

A retrospective review was conducted for all patients with molecularly confirmed *CRX*-associated retinopathy at two centres in London: Moorfields Eye Hospital and St Thomas’ Hospital. Analysis focused on patients with available ultra-widefield pseudocolour and autofluorescence imaging (Optos plc, Dunfermline, UK), and those with the specific bifocal degeneration pattern (with an additional locus in nasal retina) in both eyes were identified. Additional information collected included demographic data and molecular diagnosis from electronic patient records, and spectral-domain optical coherence tomography (SD-OCT; Heidelberg Spectralis, Heidelberg, Germany).

Visual electrophysiology performed for all 6 patients included dark-adapted (DA) and light-adapted (LA) full-field electroretinogram (ERG) and pattern electroretinogram (PERG) testing, incorporating the International Society for Clinical Electrophysiology of Vision (ISCEV) standards [7, 8], using gold foil corneal recording electrodes. Full-field ERGs were used to assess generalised rod and cone system function and pattern ERG P50 was used to assess macular function [9]. The ERG data were compared with a reference range from a group of healthy subjects (age range: 10–79 years) [10, 11]. The amplitudes of the main ISCEV Standard ERG components and b-wave to a-wave amplitude ratios were plotted as a percentage of the age-matched lower limit (5th percentile) of the reference range. Peak times were assessed as a difference from the age-matched upper limit (95th percentile) of the reference range.

The overall sex ratio in the *CRX* patient cohort was evaluated using a chi-square test. Fisher’s exact test was used to assess whether the occurrence of nasal degeneration was significantly associated with sex. Statistical significance was considered at a *p*-value of <0.05.

This study adhered to the tenets of the Declaration of Helsinki and received relevant local research ethics approval (Moorfields Eye Hospital and the Northwest London research ethics committee; 12/LO/0141). Written informed consent for genetic testing was obtained from all participating individuals.

## Results

**Fig. 1:**
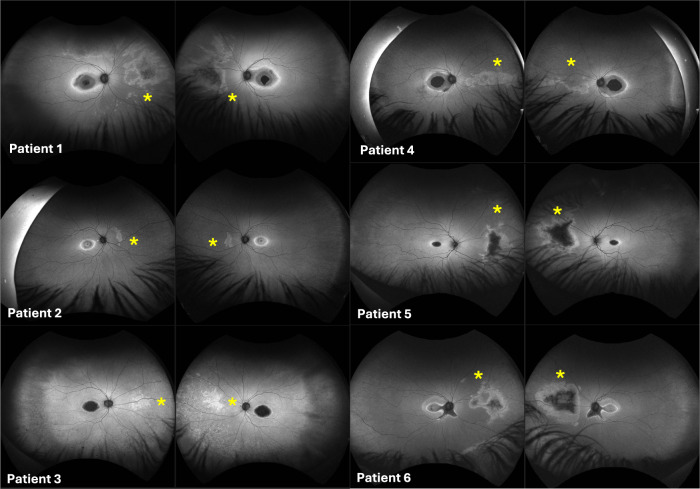
Fundus autofluorescence imaging for patients 1–6. All six patients displayed a distinct pattern of altered autofluorescence consisting of central retinal degeneration, along with an additional non-contiguous area of abnormality in the nasal periphery (marked with an asterisk).

**Fig. 2:**
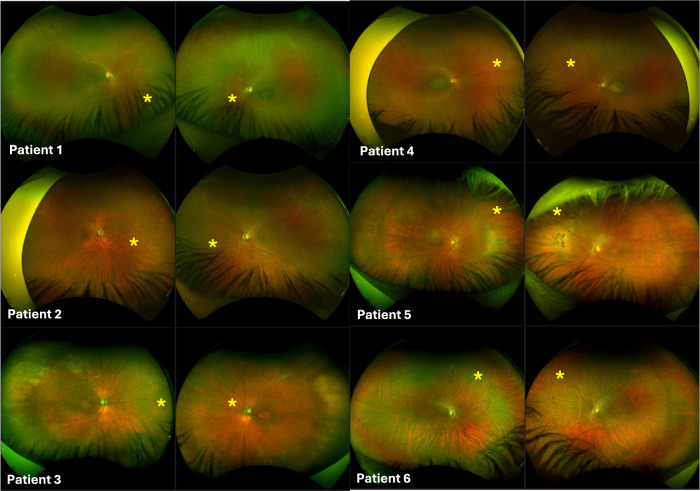
Ultra-widefield pseudocolour fundus imaging for patients 1–6. The non-contiguous nasal retinal degeneration is identifiable by an area of pigmentary alteration and pigment spicules in patients 5 and 6, but is not clearly discernible in patients 1–4. The yellow asterisk marking the area of nasal degeneration is positioned consistently in both Figs. 1, 2 for comparison.

60 patients with molecularly confirmed *CRX-*associated retinopathy from 45 families were identified. In all cases, this was linked to heterozygous *CRX* variants causing dominant disease; no individuals with biallelic *CRX* variants causing autosomal recessive disease were identified in our patient cohort. Ultra-widefield pseudocolour and autofluorescence imaging was available for 50 patients. Among these, six patients (12%), all male, displayed a distinct pattern consisting of central retinal degeneration (involving the macula or posterior pole), along with an additional area of discrete abnormality in the nasal periphery with altered autofluorescence characteristics, which was non-contiguous with the macular degeneration, and which affected a similar location in both eyes (Fig. 1). This nasal degeneration was more pronounced on autofluorescence imaging compared to clinical examination and ultra-widefield pseudocolour imaging (Fig. 2). Patient characteristics and genetic findings for these six individuals are summarised in Table 1 (with variants classified according to the American College of Medical Genetics and Genomics (ACMG) variant classification guidelines)[12].

**Table 1:** Demographic information and genetic diagnoses for patients 1–6.

Individual	Sex	Age (years)	Age of symptom onset (years)	Ethnicity	Refraction	*CRX* variant^a^	Variant type	Variant classification^b^
Patient 1 (GC5126)	Male	38	17	South Asian	OD: +0.75/−2.25 × 23° OS: +0.50/−2.00 × 168°	c.568_590del; p.(Pro190GlyfsTer38)	Frameshift	Likely pathogenic
Patient 2 (GC31119)	Male	66	54	Not stated	Not available	c.127 C > T; p.(Arg43Cys)	Missense	Pathogenic
Patient 3 (GC22925)	Male	74	50	Other	Not available	c.121 C > T; p.(Arg41Trp)	Missense	Pathogenic
Patient 4 (GC21156)	Male	49	40	Asian	OD: +0.50/−1.00 × 180°OS: −0.25/−0.50 × 150°	c.615del; p.(Ser206ProfsTer13)	Frameshift	Likely pathogenic
Patient 5 (GC22346)	Male	50	45	Not stated	OD: +4.25/−0.75 × 93°OS: +4.00/−0.75 × 91°	Whole gene deletion	Deletion	Pathogenic
Patient 6	Male	26	26	White Caucasian	Not available	c.605del; p.(Cys220SerfsTer17)	Frameshift	Likely pathogenic

^a^Corresponding to NCBI RefSeq NM_000554.6.

^b^according to the American College of Medical Genetics and Genomics (ACMG) variant classification guidelines.

*OD* right eye, *OS* left eye.

**Fig. 3:**
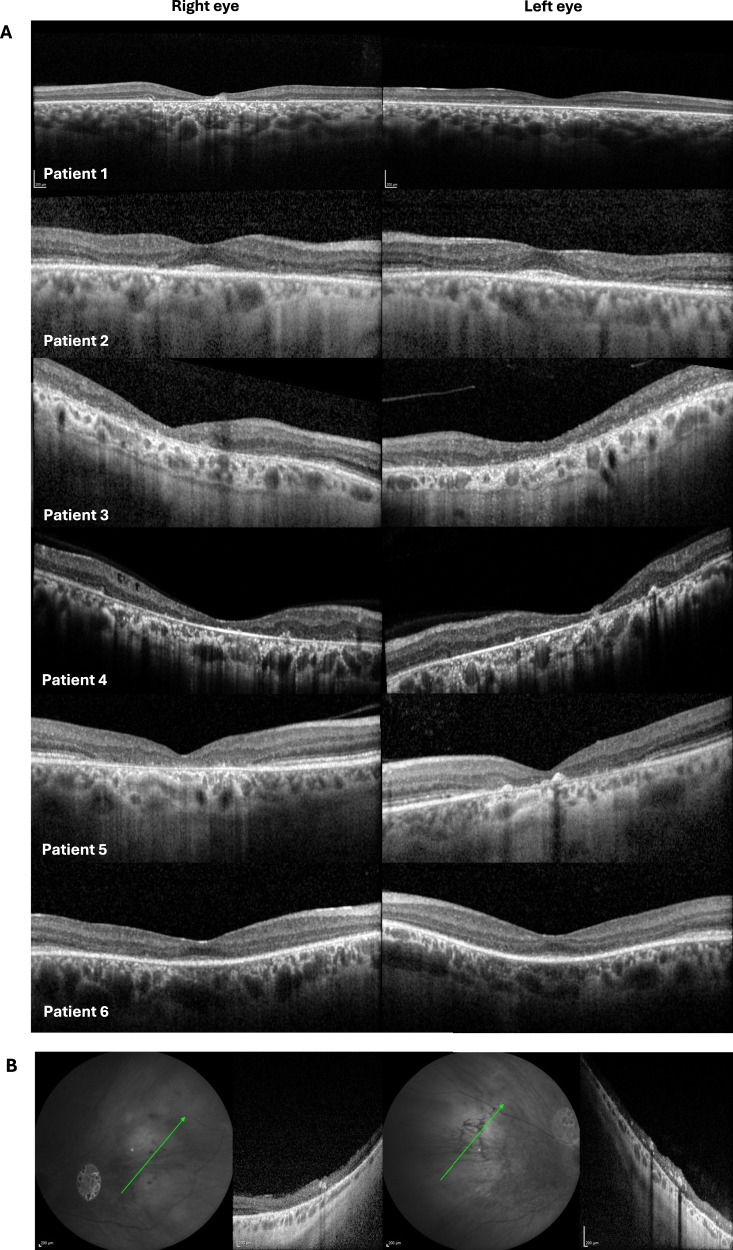
SD-OCT imaging for patients 1–6. **A** Macular SD-OCT imaging for patients 1–6 showing central outer retinal disruption; **B** SD-OCT through the right and left nasal areas of retinal degeneration in patient 5, showing retinal disorganization.

Figure 3 depicts macular SD-OCT findings in all 6 patients; in one patient (Patient 5), SD-OCT imaging was also performed through the nasal area of retinal degeneration and showed loss of retinal structure and lamination throughout this region.

There was a high degree of inter-ocular ERG symmetry based on the ISCEV Standard DA 0.01, DA 10 ERG a- and b-waves, LA 30 Hz ERG and LA 3 (single flash) ERG a- and b-wave amplitudes (slope = 0.94; *r*^2^ = 0.97) and peak times (slope = 0.99; *r*^2^ = 0.99).

**Fig. 4:**
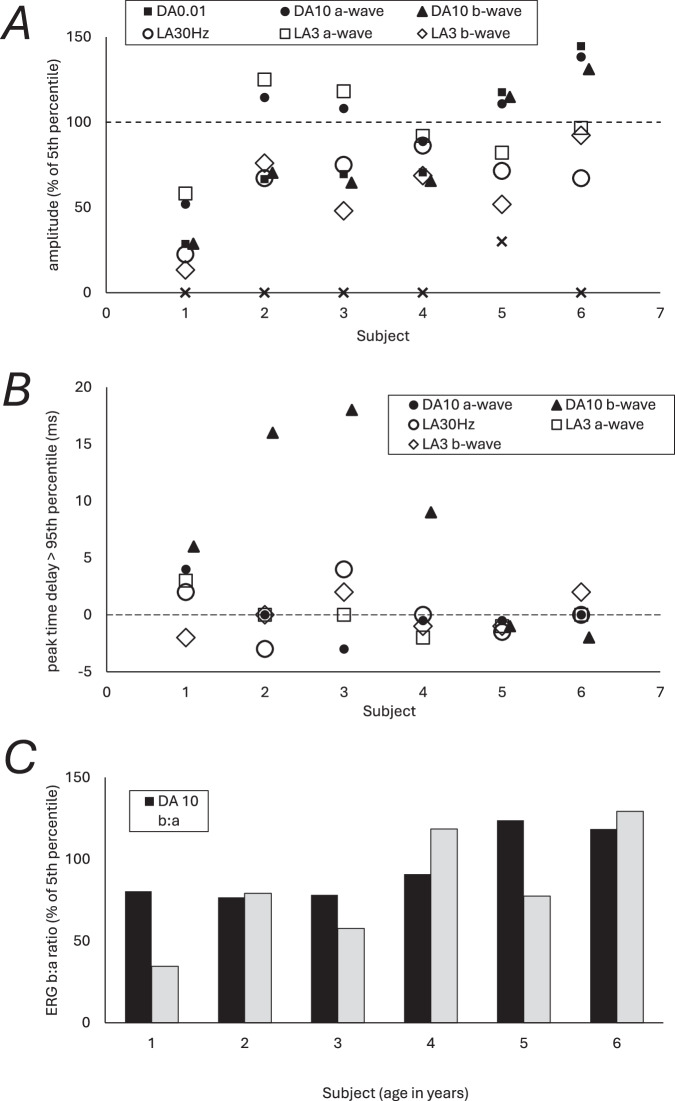
Electrodiagnostic findings for patients 1-6. Pattern and full-field ERG findings summarised in patients 1–6 tested according to ISCEV standard methods. **A** The amplitudes of the DA0.01 ERG, DA 10 ERG a- and b-waves, LA 30 Hz ERG and LA 3 ERG a- and b-wave are plotted as a percentage of the age-matched 5th percentile of the (“normal”) reference range. **B** The peak times of the main ERG components are plotted as a difference compared with the 95th percentile of the reference range. **C** The DA10 ERG and LA3 ERG b:a ratios are compared with age-associated reference values specific to the stimulus system used (5th percentiles of b:a ratio 1.1–1.2 for the DA10 ERGs; 2.9–3.1 for the LA3 ERGs). Broken lines indicate the limits of normality. Note that subject-specific values for the DA 10 ERG b-waves in (**A**) and (**B**) are plotted slightly laterally for clarity.

The PERG P50 and full-field ERG findings and patient ages at the time of testing are summarised in Fig. 4. PERG P50 was bilaterally subnormal in all cases, including 5 of 6 with bilaterally undetectable responses, consistent with severe macular dysfunction. Comparison of DA and LA ERGs (summarised in Fig. 4) revealed generalised retinal involvement confined to the cone system (*n* = 2), cone more than rod system dysfunction (*n* = 3) or similar severity of cone and rod system involvement (*n* = 1; subject 4). The DA 10 ERG b-waves were markedly delayed in 4 of 6 cases, including one with additional a-wave delay (patient 1); the LA ERGs showed relatively mild (*n* = 3) or borderline (*n* = 2) delay or were of normal timing (*n* = 1). The LA 3 (single flash) ERG b:a ratio was subnormal in 5 cases, and the DA 10 ERG b:a ratio subnormal in 4, in keeping with a possible locus of dysfunction post-phototransduction. In two of those with a low DA10 ERG b:a ratio, there was additional moderate (*n* = 1; patient 1) or mild (*n* = 1; patient 4) a-wave reduction, consistent with additional rod photoreceptor involvement.

## Discussion

In this study, we present data from six patients with *CRX*-associated retinopathy in whom a bifocal pattern of degeneration was discernible in both eyes: in each eye, there was a discrete area of altered autofluorescence in the nasal periphery that was not contiguous with the degeneration affecting the central retina. Bifocal retinal lesions have been described in progressive bifocal chorioretinal atrophy, a rare, early-onset chorioretinal dystrophy linked to a non-coding variant upstream of *PDRM13* [13]. Our findings show that a proportion of patients with *CRX*-associated retinopathy (12% in our study) also exhibit bifocal areas of retinal degeneration, albeit with a distinct appearance. Electrophysiology was consistent with a cone or cone-rod pattern of dysfunction, with a possible locus that was post-phototransduction (Fig. 4c), as has been previously reported in *CRX*-associated disease [14–16].

The precise mechanism underlying this discrete nasal retinal degeneration remains uncertain. Some forms of inherited retinal disease show sectoral retinal pigmentary changes with a predilection for inferior and nasal involvement; this phenomenon is commonly associated with pathogenic *RHO* variants but has also been noted in connection with other genes [17]. In these cases, given the inferior retinal predilection, light exposure has been proposed as a contributing factor. It has also been hypothesised that gradients of gene expression in the retina may predispose certain retinal quadrants differently; further investigation is warranted to explore these potential mechanisms.

Interestingly, while the sex ratio with our overall *CRX* patient cohort is not significantly skewed (34 male 26 female; *p* > 0.05), it is notable that all six patients identified with nasal degeneration in our study were male (*p* = 0.031). Significant sex imbalances have been observed in certain autosomally-inherited macular dystrophies (such as those associated with *ABCA4*, *BEST1* and *EFEMP1*),[18, 19] although the underlying mechanisms remain unclear. Messenger RNAs for oestrogen, progesterone, and androgen receptors have been detected in retinal cells [20, 21], and it may be that sex-specific hormonal differences may act as modifying factors influencing disease presentation.

We did not observe a specific genotype-phenotype correlation in our study; the presence of nasal degeneration did not correlate with any variant type, and was seen across ethnicities. Among the six patients in our series, a spectrum of disease variants was identified; two had missense variants, three had frameshift variants, and one harboured a large multi-gene deletion involving *CRX*, *TPRX1* and *SULT2A1* (Table 1). This deletion has recently been associated with a dominant late-onset macular disease [4], although none of the 8 patients reported in that study showed signs of nasal degeneration. Intra-familial variability was also observed; in one case (Patient 1), both the affected sister and father, despite sharing the same *CRX* disease allele, did not exhibit any signs of nasal degeneration, suggesting the potential influence of trans-acting or environmental modifiers.

Several limitations of our study should be acknowledged. The prevalence estimate should be interpreted in light of the small cohort size, reflecting the rarity of *CRX*-associated retinopathy. It is possible that some patients who did not initially exhibit nasal degeneration may go on to develop this over time; however, given that this feature was absent in some individuals with long disease durations exceeding 20 years, it does not appear to be an inevitable development in all cases of dominant *CRX*-associated retinopathy. Additionally, some patients have been noted to have areas of nasal degeneration that were contiguous with the more central degeneration; it is possible that these areas were previously non-contiguous and later became confluent over time. Furthermore, OCT imaging of the nasal retina is not routinely performed, and it is possible that some patients may have had nasal degeneration detectable by OCT, but not by autofluorescence imaging. Finally, OCT imaging through the nasal area of retinal degeneration was only available for one of the six patients in the study, and it remains uncertain if the OCT findings will be replicated in the other cases; future studies could investigate this more systematically.

There have been significant advances in genomic testing for inherited retinal disease in the UK, due to the success of initiatives including the 100,000 Genomes Project and, more recently, the NHS Genomic Medicine Service [22, 23]. Despite this progress, achieving a molecular diagnosis remains challenging, with 40–50% of cases remaining unresolved [24, 25], due in part to difficulties with variant identification and interpretation. Recognising distinctive phenotypes, such as the distinct pattern of nasal degeneration observed in some cases of *CRX*-associated retinal disease, which is not typically seen in other forms of inherited retinal disease, is important. This can aid in guiding genetic investigations and facilitate the identification of potentially pathogenic gene variants associated with specific phenotypic presentations. Given the autosomal dominant inheritance and phenotypic variability characteristic of *CRX*-associated retinopathy, obtaining a molecular diagnosis is particularly important for effective family counselling and access to emerging therapies [26, 27].

## Summary

### What was known before

- Pathogenic *CRX* variants are associated with various retinal dystrophy phenotypes, including macular dystrophy, cone-rod dystrophy, and Leber congenital amaurosis.
- Dominant *CRX*-associated retinopathy displays significant phenotypic variability.

### What this study adds

- A proportion of patients with *CRX*-associated retinopathy (12% in this study) exhibit bifocal areas of retinal degeneration, consisting of central retinal degeneration along with an additional discrete non-contiguous affected area in the nasal periphery with altered autofluorescence characteristics. This distinct pattern is not typically seen in other forms of inherited retinal disease.
- Recognising distinct phenotypes can guide interpretation of genetic data towards potentially pathogenic variants in genes associated with the specific phenotypic presentation, facilitating personalised clinical care.

## Data Availability

Data supporting the findings of this study are available from the study authors on request.
